# Supplementary material for: Native Bovine Hydroxyapatite Powder, Demineralised Bone Matrix Powder, and Purified Bone Collagen Membranes Are Efficient in Repair of Critical-Sized Rat Calvarial Defects
Source: Materials (Basel). 2020 Jul 31;13(15):3393. doi: 10.3390/ma13153393 (PMC7436118; doi:10.3390/ma13153393)
Supplement: Supplementary file 1 [file materials-13-03393-s001.pdf]

materials

Supplementary Materials

Native Bovine Hydroxyapatite Powder, Demineralised Bone Matrix Powder, and Purified Bone Collagen Membranes are Efficient in Repair of Critical-Sized Rat Calvarial Defects

Alexey Veremeev, Roman Bolgarin, Vladimir Nesterenko, Alexander Andreev-Andrievskiy and Anton Kutikhin

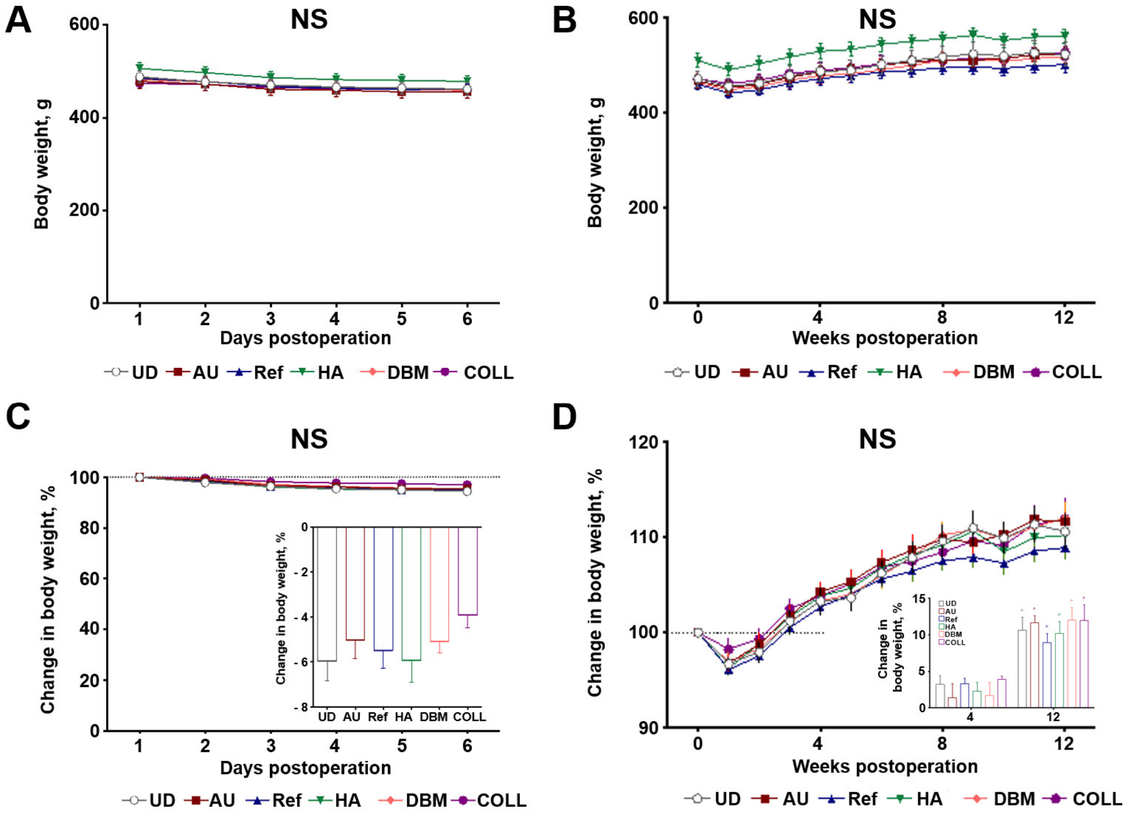

Figure S1. General safety examination of the implants. (**A**) Daily serial measurements of body weight during the first week postimplantation; (**B**) weekly serial measurements of body weight during 12 weeks postimplantation; and (**C**) daily serial calculation of body weight increment/decrement during the first week postimplantation. Inserts show relative change in body weight across the groups at 6 days postoperation; (**D**) weekly serial calculation of body weight increment/decrement during 12 weeks postimplantation. Inserts show relative change in body weight across the groups at 4 and 12 weeks postoperation. Unfilled defect, autograft, and Geistlich Bio-Oss® represent a negative control, positive control, and reference product, respectively. $N = 12$ animals per group, two-way analysis of variance with Tukey's multiple comparisons test, no statistically significant differences. NS—non-significant intergroup differences, UD—unfilled defect, AU—autograft, Ref—reference product (Geistlich Bio-Oss®), HA—refined hydroxyapatite powder, DBM—demineralised bone matrix powder, COLL—purified bone collagen membranes.

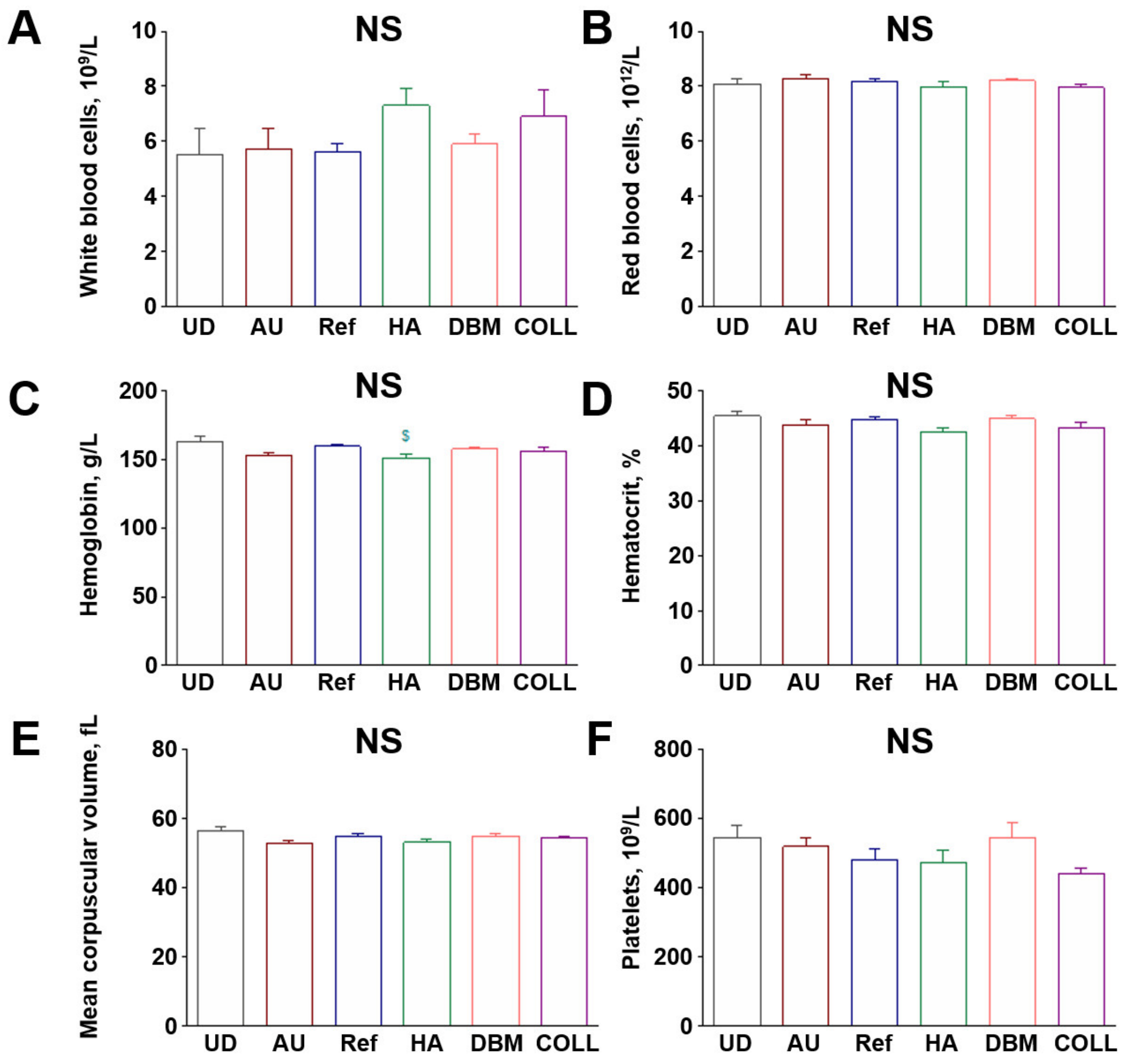

Figure S2. Complete blood count measured at 12 weeks postimplantation. (**A**) White blood cell count; (**B**) red blood cell count; (**C**) haemoglobin measurement; (**D**) haematocrit calculation; (**E**) mean corpuscular volume measurement; and (**F**) platelet count. Unfilled defect, autograft, and Geistlich Bio-Oss® represent a negative control, positive control, and reference product, respectively. $N = 6$ animals per group, one-way analysis of variance with Tukey's multiple comparisons test, no statistically significant differences. NS—non-significant intergroup differences, UD—unfilled defect, AU—autograft, Ref—reference product (Geistlich Bio-Oss®), HA—refined hydroxyapatite powder, DBM—demineralised bone matrix powder, COLL—purified bone collagen membranes.

Table S1. Biochemical analysis performed at 12 weeks postimplantation.

Parameter	UD	AU	Ref	HA	DBM	COLL	Ref Range	*p*
Total protein (g/dL)	7.11 ± 0.23	6.99 ± 0.09	6.92 ± 0.08	6.90 ± 0.12	6.91 ± 0.19	6.96 ± 0.11	6.31–7.71	0.9
Albumin (g/dL)	4.22 ± 0.11	4.10 ± 0.07	4.16 ± 0.05	4.14 ± 0.05	4.13 ± 0.08	4.12 ± 0.07	3.52–4.27	0.9
Globulins (g/dL)	2.90 ± 0.15	2.90 ± 0.08	2.75 ± 0.06	2.76 ± 0.08	2.79 ± 0.17	2.84 ± 0.09	2.64–3.53	0.9
Glucose (mmol/L)	11.1 ± 0.6	13.8 ± 1.4	12.2 ± 1.7	11.4 ± 0.7	12.3 ± 0.7	11.0 ± 0.9	4.3–7.81	0.5
Triglycerides (mmol/L)	2.00 ± 0.22	1.87 ± 0.31	1.45 ± 0.06	2.02 ± 0.15	2.20 ± 0.24	2.05 ± 0.12	0.67–2.15	0.2
Cholesterol (mmol/L)	3.25 ± 0.17	3.06 ± 0.18	2.96 ± 0.13	3.29 ± 0.26	3.51 ± 0.42	3.41 ± 0.26	2.17–3.00	0.6
Urea (mmol/L)	6.41 ± 0.41	6.24 ± 1.14	6.48 ± 0.54	7.34 ± 0.50	6.33 ± 0.90	4.85 ± 0.39	6.46–8.93	0.2
Creatinine (μmol/L)	87 ± 4	79 ± 6	86 ± 10	74 ± 13	61 ± 6	75 ± 5	–	0.3
Bilirubin (μmol/L)	2.31 ± 1.24	2.19 ± 1.02	3.31 ± 0.56	4.15 ± 1.12	3.65 ± 1.98	3.13 ± 0.71	–	0.8
Uric acid (μmol/L)	207 ± 26	200 ± 41	286 ± 61	264 ± 40	234 ± 32	196 ± 30	–	0.5
LDH (IU/L)	470 ± 69	522 ± 150	838 ± 250	771 ± 115	486 ± 110	564 ± 109	–	0.3
AP (IU/L)	196 ± 12	240 ± 24	195 ± 20	215 ± 12	213 ± 13	162 ± 39	56–145	0.3
ALT (IU/L)	41 ± 8	53 ± 5	64 ± 12	66 ± 5	53 ± 12	44 ± 10	20–81	0.3
AST (IU/L)	153 ± 28	78 ± 29	62 ± 15	91 ± 16	134 ± 34	95 ± 29	41–115	0.2

N = 6 animals per group, one-way analysis of variance with Tukey's multiple comparisons test, no statistically significant differences. UD—unfilled defect, AU—autograft, Ref—reference product (Geistlich Bio-Oss®), HA—refined hydroxyapatite powder, DBM—demineralised bone matrix powder, COLL—purified bone collage membranes, LDH—lactate dehydrogenase, AP—alkaline phosphatase, ALT—alanine aminotransferase, AST—aspartate aminotransferase, IU—international units.

© 2020 by the authors. Submitted for possible open access publication under the terms and conditions of the Creative Commons Attribution (CC BY) license (http://creativecommons.org/licenses/by/4.0/).
